# Identifying critically ill children in Malawi: A modified qSOFA score for low-resource settings

**DOI:** 10.1371/journal.pgph.0002388

**Published:** 2024-01-25

**Authors:** Mercy Kumwenda, Roxanne Assies, Ilse Snik, Gloria Chatima, Josephine Langton, Yamikani Chimalizeni, Sam T. Romaine, Job B.M. van Woensel, Philip Pallmann, Enitan D. Carrol, Job C.J. Calis

**Affiliations:** 1 Department of Paediatrics and Child Health, Kamuzu University of Health Sciences, Blantyre, Malawi; 2 Department of Paediatrics and Child Health, Kamuzu Central Hospital, Lilongwe, Malawi; 3 Department of Global Health and PICU, Amsterdam Institute for Global Health and Development and Emma Children’s Hospital, Amsterdam UMC, Location University of Amsterdam, Amsterdam, the Netherlands; 4 Amsterdam Public Health, Global Health and Quality of Care, Amsterdam, the Netherlands; 5 Institute of Infection, Veterinary and Ecological Sciences, University of Liverpool, Liverpool, United Kingdom; 6 Centre for Trials Research, College of Biomedical and Life Sciences, Cardiff University, Cardiff, United Kingdom; Baylor College of Medicine, UNITED STATES

## Abstract

In low-resource settings, a reliable bedside score for timely identification of children at risk of dying, could help focus resources and improve survival. The rapid bedside Liverpool quick Sequential Organ Failure Assessment (LqSOFA) uses clinical parameters only and performed well in United Kingdom cohorts. A similarly quick clinical assessment-only score has however not yet been developed for paediatric populations in sub-Saharan Africa. In a development cohort of critically ill children in Malawi, we calculated the LqSOFA scores using age-adjusted heart rate and respiratory rate, capillary refill time and Blantyre Coma Scale, and evaluated its prognostic performance for mortality. An improved score, the Blantyre qSOFA (BqSOFA), was developed (omitting heart rate, adjusting respiratory rate cut-off values and adding pallor), subsequently validated in a second cohort of Malawian children, and compared with an existing score (FEAST-PET). Prognostic performance for mortality was evaluated using area under the receiver operating characteristic curve (AUC). Mortality was 15.4% in the development (N = 493) and 22.0% in the validation cohort (N = 377). In the development cohort, discriminative ability (AUC) of the LqSOFA to predict mortality was 0.68 (95%-CI: 0.60–0.76). The BqSOFA and FEAST-PET yielded AUCs of 0.84 (95%-CI:0.79–0.89) and 0.83 (95%-CI:0.77–0.89) in the development cohort, and 0.74 (95%-CI:0.68–0.79) and 0.76 (95%-CI:0.70–0.82) in the validation cohort, respectively. We developed a simple prognostic score for Malawian children based on four clinical parameters which performed as well as a more complex score. The BqSOFA might be used to promptly identify critically ill children at risk of dying and prioritize hospital care in low-resource settings.

## Introduction

Globally, paediatric mortality has decreased over the last decade, although over 5 million children died in 2020, of which the highest burden was reported in sub-Saharan Africa [1]. Most of these children suffered from treatable diseases, such as gastroenteritis, pneumonia, and malaria, yet hospital mortality in low- and middle-income countries has not decreased [1,2]. Children often present to hospital late, with severe disease, and most deaths occur within 48 hours of admission [2].

Early recognition of critical illness is essential to initiate prompt treatment, allocate resources and improve survival of hospitalized children. However, identifying these critically ill children in hospitals with limited resources presents specific challenges. Technological support and specialized paediatric staff to ensure intensive monitoring may be lacking, which is particularly relevant in critically ill children who may show minimal clinical signs and experience rapid disease progression [3]. If children at highest risk of dying could be identified in time, reliably, and without additional resources, this would help clinicians who could then initiate appropriate and timely life-saving treatment.

The Emergency Triage Assessment and Treatment (ETAT) guidelines, introduced by the World Health Organization (WHO), reduced acute paediatric deaths when implemented in low-resource settings [4,5]. ETAT is used at initial screening upon entry to a health facility. After this initial screening, there is no routinely used score that could help clinicians identify children most at risk of dying and in need of critical care, despite a large body of previous studies combining clinical parameters of respiratory, circulatory and neurological status. Previous studies have also validated, adapted, or developed early warning scores specifically for low-resource settings (including SICK, PEWS-RL, LODS, PEDIA, FEAST-PET). These scores, however, require monitoring or laboratory technologies, are complex to use as they consist of many parameters, or have been developed for a specific population or to detect clinical deterioration in children already admitted [6–12]. The FEAST-Paediatric Emergency Triage (FEAST-PET) was developed for use in the Emergency Department without the need for laboratory testing or monitoring equipment, however it is a semi-complex score with 11 clinical components.

The quick Sequential Organ Failure Assessment (qSOFA), a bedside composite score, has been developed for early recognition of adults with suspected sepsis and increased risk of in-hospital mortality [13]. This score has been adapted to the Liverpool qSOFA (LqSOFA) which performed better than the original qSOFA in predicting critical care admission and mortality in febrile children in the UK [14]. The LqSOFA could be very useful in low-resource settings, for it consists of easy- and quick-to-score clinical parameters (heart rate, respiratory rate, capillary refill time, consciousness level) and no laboratory tests. The LqSOFA showed promising results in a primary care setting with limited resources in Thailand [15].

The overall aim of this study was to develop and validate an accurate and simple bedside score to identify critically ill children with the highest risk of death presenting to a hospital with limited resources. We specifically aimed to, a) evaluate the prognostic accuracy of the LqSOFA in a cohort of critically ill Malawian children presenting to the paediatric emergency department, b) adjust the score to better fit this setting which we named the Blantyre qSOFA (BqSOFA), c) validate the BqSOFA in a second cohort of Malawian children who were critically ill upon admission and d) compare its performance with another validated bedside score, the FEAST-PET [16]. We compared the BqSOFA with the FEAST-PET as both scores were developed for low-resource emergency settings, combining vital parameters that can be measured using clinical judgement only [16].

## Methods

### Study setting

This study includes critically ill children upon admission to Queen Elizabeth Central Hospital (QECH), a tertiary referral hospital in Blantyre, Malawi where approximately 12,000 patients are admitted through the paediatric emergency department every year. Critically ill children are admitted to High Dependency Units (HDU) where up to 40 children can be admitted, with more human and technical resources, however at the time of this study, without access to continuous monitoring. From 2018, a six-bedded Paediatric Intensive Care Unit (PICU) was opened.

### Participants

Two cohorts of critically ill children admitted to QECH provided the data for this study. The *development cohort* included 493 children aged two months to 16 years with respiratory distress and/or impaired consciousness and at least one sign of shock upon admission to the emergency department. Clinical and outcome data were collected from admission forms in a prospective observational study between 1 February 2019 and 31 January 2020. Twelve (2.4%) children were excluded from the original dataset (N = 505) due to missing outcome data. The *validation cohort* included 377 children aged two months to 16 years with possible pneumonia or meningitis. Data were prospectively collected as part of an observational study between April 2004 and October 2006. For details, we refer to the original papers, Table A and Fig A in S1 Text [17,18].

### Predictors and outcome

The LqSOFA consists of four clinical parameters; age-adjusted heart rate (HR), age-adjusted respiratory rate (RR), capillary refill time (CRT), and consciousness level (AVPU, Table B in S1 Text) [14]. The predictor values for the development cohort were the first measured in the emergency department, and for the validation cohort, these were the most abnormal values measured in the first four hours of admission to HDU.

To calculate the LqSOFA, each component scored zero if normal or one if abnormal, resulting in a maximum score of four. HR and RR above the age-adjusted 99^th^-percentile (Bonafide et al.) were considered abnormal [14,19]. In Malawi, the Blantyre Coma Scale (BCS, range 0–5) was used instead of AVPU; a BCS < 5 was considered abnormal [20].

To improve the LqSOFA we compared cut-off values in the development cohort using other published cut-off values for low-resource settings, as well as defining cut-off values based on the study population age-specific 90^th^-percentile (Table C and Fig B in S1 Text). We selected possible additional clinical predictors (pallor, dehydration, nutritional status) based on previous literature, clinical reasoning and routine use by clinicians as they are included in the ETAT guidelines [4,16,21,22]. Outcome was in-hospital mortality.

The FEAST-PET consists of eight clinical parameters and was developed in a sub-Saharan African setting [16]. In both cohorts, one of the eight variables (lung crepitations in the development cohort, weak radial pulse in the validation cohort) needed for the FEAST-PET was not present in the database (Table D in S1 Text).

### Statistical analysis

Analysis was performed in SPSS (IBM, v26) and R (v4.1.0). In both datasets, we excluded cases in which outcome data or two or more score parameters were missing (Fig A in S1 Text). If one parameter was missing, this was assumed normal [14].

We first calculated the LqSOFA in the development cohort and evaluated the prognostic performance. We performed sensitivity analyses by comparing area under the receiver operating characteristic curve (AUC) when the missing values were scored as normal or as abnormal, to evaluate if this would skew our results, which was not the case (AUC: 0.66, 95%-CI: 0.58–0.73 versus AUC: 0.67, 95%-CI: 0.60–0.75). Discriminative performance was assessed by calculating the AUC, sensitivity, specificity, negative and positive predictive value (NPV, PPV). As a measure of calibration, we calculated the proportion of children who died for the different LqSOFA values.

We then amended the LqSOFA to the BqSOFA. Possible additional predictors were identified by assessing crude associations with outcome using the χ^2^-test (Fisher’s exact test if small sample). A two-sided p-value<0.05 was considered statistically significant. We amended the LqSOFA by substituting, adding, and removing different predictors and evaluating these modifications with the above measures of discriminative performance and net reclassification index (NRI) in a stepwise approach [23]. NRI, a method to compare changes in discriminative ability after changing the parameters, was used to test if the new model improved prediction of mortality. This resulted in the BqSOFA (Table E in S1 Text).

We evaluated the performance of the BqSOFA and LqSOFA in the validation cohort. Finally, we compared the prognostic accuracy of the BqSOFA and LqSOFA with the FEAST-PET in both cohorts. We reported results following the TRIPOD-guidelines [24].

### Ethics statement

Both studies were granted required approval from the College of Medicine Research and Ethics Committee (COMREC), Malawi [17,18]. The validation cohort (original) study was granted additional approval by The Liverpool School of Tropical Medicine Research Ethics Committee [17]. Parents or guardians of included children gave written informed consent in both studies. Additional information regarding the ethical, cultural, and scientific considerations specific to inclusivity in global research is included in the Supporting Information.

## Results

### Study population

Mortality was 15.4% in the development cohort and 22.0% in the validation cohort. Median age was 17.0 and 26.7 months, respectively (Table 1). In the development cohort, children were mainly diagnosed with viral/reactive respiratory disease (40.4%), severe pneumonia (14.3%), and malaria (12.6%). Sepsis was suspected in 5.7% of cases. In the validation cohort, children were diagnosed with meningitis (74.8%) or pneumonia (25.2%).

**Table 1:** Descriptive data of the development and validation cohort.

	Development cohort (N = 493) n/N (%)	Validation cohort (N = 377) n/N (%)	
**Characteristics**			
Age (months) median (IQR)	17.0 (9.0–36.0)	26.7 (8.2–74.0)	
Male	277/486^a^ (57.0)	215/377 (57.0)	
Fever or hypothermia (history of fever/ axillary temperature ≥38.0 ^o^C/ axillary temperature ≤36.0 ^o^C)	401/491^a^ (81.7)	262/368^a^ (71.2)	
Positive malaria test (smear or rapid test)	65/383^a^ (17.0)	13/360^a^ (3.6)	
Anaemia (haemoglobin <10g/dL)	146/324^a^ (45.1)	260/364^a^ (71.4)	
HIV positive	27/352^a^ (7.7)	190/376^a^ (50.5)	
**Main diagnosis on discharge**			
Viral/reactive respiratory diseases	190/470^a^ (40.4)	Meningitis:	282/377 (74.8)
Severe pneumonia	67/470^a^ (14.3)	Pneumonia:	95/377 (25.2)
Gastroenteritis	53/470^a^ (11.3)		
Malaria	59/470^a^ (12.6)		
Presumed sepsis	27/470^a^ (5.7)		
**Outcome**			
In-hospital mortality	76/493 (15.4)	83/377 (22.0)	
**Predictors**			
*Respiratory*			
Increased respiratory rate (>99^th^ percentile Bonafide et al.)	121/289^a^ (41.9)	150/374^a^ (40.1)	
Increased respiratory rate (>90^th^ percentile development cohort)	33/289^a^ (11.4)	37/374^a^ (9.9)	
Respiratory distress	406/492^a^ (82.5)	139/371^a^ (37.5)	
Lung crepitations	*Missing* ^*b*^	107/372^a^ (28.8)	
*Circulatory*			
Increased heart rate (>99^th^ percentile Bonafide et al.)	284/480^a^ (59.2)	95/367^a^ (25.9)	
Increased heart rate (>90^th^ percentile development cohort)	61/480^a^ (12.7)	28/367^a^ (7.6)	
Capillary refill time >3 seconds^c^	70/445^a^ (15.7)	153/369^a^ (41.5)	
Weak radial pulse	47/90^a^ (52.2)	*Missing*	
Dehydration (bedside, moderate or severe)	71/472^a^ (15.0)	68/371^a^ (18.3)	
Pallor (bedside, moderate or severe)	96/476^a^ (20.2)	20/361^a^ (5.5)	
*Neurological*			
Blantyre Coma Score <5	117/473^a^ (24.7)	196/377 (52.0)	
*Other*			
Poor nutritional status^d^	39/459^a^ (8.5)	68/376^a^ (18.1)	
**Scores**			
LqSOFA >2 variables missing	41/493 (8.3)	2/377 (0.5)	
BqSOFA >2 variables missing	40/493 (8.1)	0/377 (0)	
FEAST-PET >2 variables missing	87/493 (17.6)	13/377 (3.4)	

^a^ Not all data available for this variable: Denominator indicates for how many children of the total population this variable was available.

^b^ Not included in the development cohort dataset. This study had a different study aim, and in this set-up lung crepitations were not included in the Case Record Form of this study.

^c^ The WHO ETAT definition (for shock, CRT >3 seconds) was used for the development cohort. For the validation cohort with septic patients the original LqSOFA scoring was used: CRT >3 seconds.

^d^ Quick assessment through eyeballing nutritional status in development cohort. In the validation cohort mid upper arm circumference (MUAC) <12.5cm was used.

### LqSOFA

**Fig 1:**
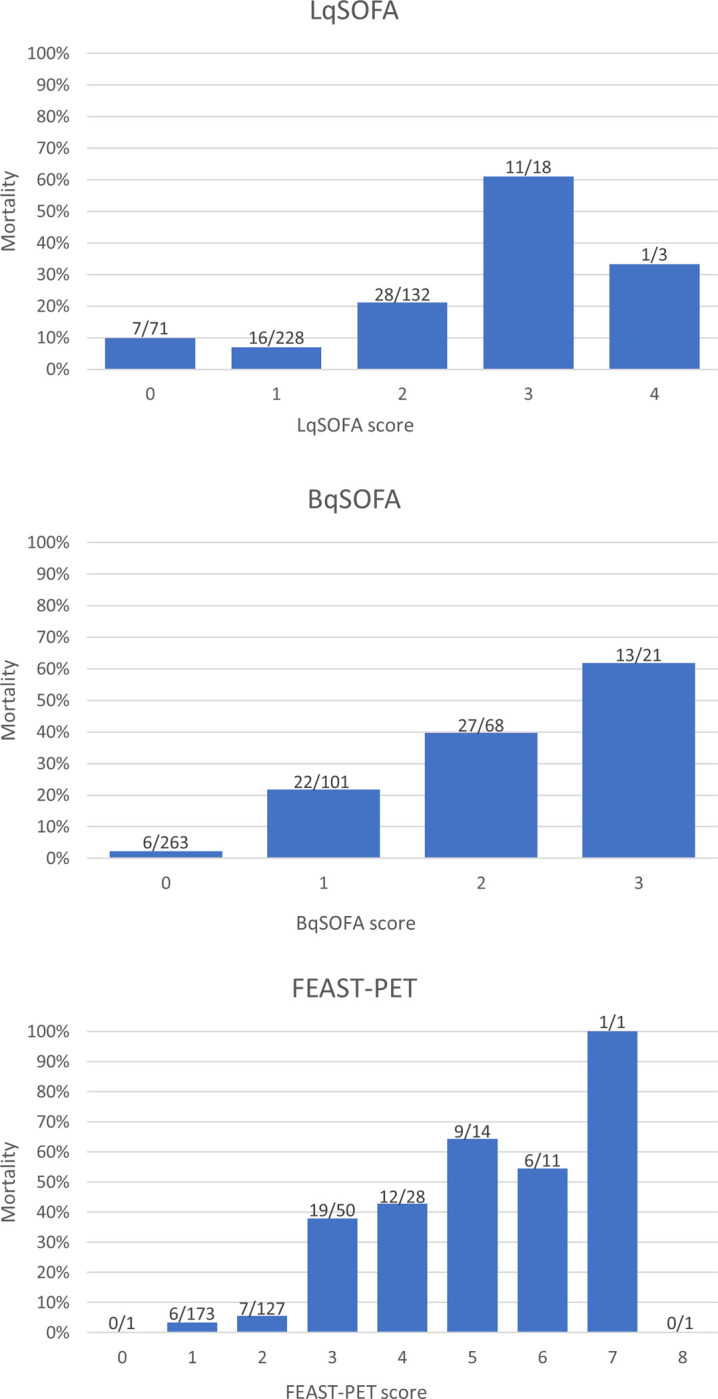
Bar charts of LqSOFA, BqSOFA and FEAST-PET in the development cohort.

**Fig 2:**
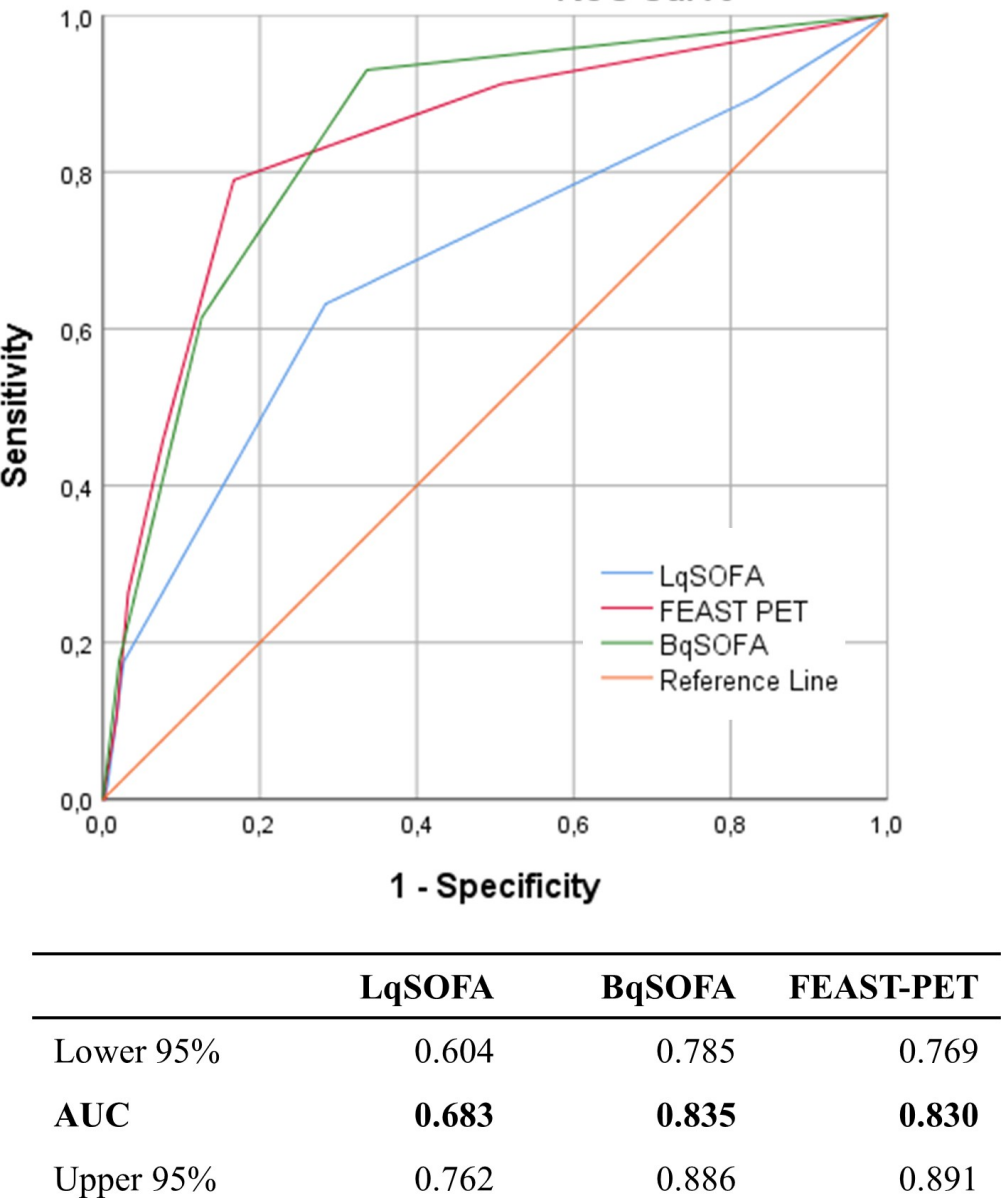
Area under receiver operating characteristic curve (AUC) for each score for predicting mortality in the development cohort.

We first evaluated the prognostic accuracy of the LqSOFA in the development cohort. For 452 (91.7%) children the LqSOFA could be calculated, and 381 (84.3%) had a score of >1 (Table 1). Mortality varied for the different score values. 7/71 (9.9%) children who scored zero and 16/228 (7.0%) children who had a LqSOFA score of one died (Fig 1). Discriminative ability (AUC) to predict death was 0.68 (95%-CI: 0.60–0.76) (Fig 2). Table 2 shows the prognostic performance of the LqSOFA for different cut-offs.

**Table 2:** Prognostic performance of LqSOFA, BqSOFA and FEAST-PET score in development cohort.

Score	Sensitivity % (95%-CI)	Specificity % (95%-CI)	PPV^1^ % (95%-CI)	NPV^2^ % (95%-CI)
LqSOFA ≥1	88.9 (77.8–95.0)	16.5 (13.0–20.6)	14.7 (11.4–18.7)	90.1 (80.2–95.6)
LqSOFA ≥2	63.5 (50.4–75.0)	71.0 (66.1–75.4)	26.1 (19.5–34.0)	92.3 (88.5–95.0)
LqSOFA ≥3	19.0 (10.6–31.3)	97.7 (95.5–98.9)	57.1 (34.4–77.4)	88.2 (84.6–91.0)
BqSOFA >1	91.2 (81.1–96.4)	66.8 (61.8–71.4)	32.6 (26.1–39.9)	97.7 (94.9–99.1)
BqSOFA >2	58.9 (46.2–70.4)	87.3 (83.4–90.4)	44.9 (34.5–55.8)	92.3 (88.9–94.7)
BqSOFA >3	19.1 (11.0–30.8)	97.9 (95.8–99.0)	61.9 (38.7–81.0)	87.3 (83.7–90.2)
FEAST-PET >1	100 (92.5–100)	0.3 (0.02–1.9)	14.8 (11.6–18.7)	100 (5.5–100)
FEAST-PET >2	90.0 (78.8–95.9)	48.6 (43.2–54.0)	23.3 (18.1–29.4)	96.6 (92.3–98.6)
FEAST-PET >3	78.3 (65.5–87.5)	83.2 (78.8–86.9)	44.8 (35.2–54.8)	95.7 (92.5–97.6)
FEAST-PET >4	46.7 (33.9–59.9)	92.2 (88.7–94.7)	50.9 (37.2–64.5)	90.9 (87.3–93.6)

^1^ Positive predictive value.

^2^ Negative predictive value.

### Stepwise development of Blantyre qSOFA (BqSOFA)

As the LqSOFA had moderate discriminative ability, we amended this score to improve prognostic accuracy for mortality in a Malawian setting. Univariate analysis of clinical predictors showed that increased HR was not associated with in-hospital mortality. Children with an increased HR (>99^th^ percentile Bonafide et al.) had a lower mortality compared to children with a normal or lower HR (<99^th^ percentile Bonafide et al., 7.9% vs. 21.9%, p<0.001). There was a similar trend when 90^th^ percentile development cohort cut-offs were used for HR. Increased RR was not associated with mortality when using the >99^th^ percentile Bonafide, yet when using the >90^th^ percentile of the development cohort this was associated with increased mortality (p = 0.017). Table F in S1 Text describes univariate analysis of the different variables we considered substituting, adding to, or removing from the LqSOFA.

Substituting the variables HR and RR in the LqSOFA with 90^th^ percentile of our development cohort resulted in a score with an AUC of 0.79 (95% CI: 0.73–0.86). As the HR (90^th^ percentile) did not show a strong association with mortality, we removed HR from the score. This resulted in a modified score with three variables: RR (90^th^ percentile), CRT, and BCS, of which the AUC was 0.82 (95%-CI: 0.76–0.88). The NRI of this modified LqSOFA compared to the previous model for cut-off values >1 and >2 was 8.18% (95%-CI: 3.79–12.58) and -0.86% (95%-CI: -5.51–3.79), respectively.

Additional predictors of mortality considered in the univariate analysis were dehydration, poor nutritional status, and pallor. Adding each of these to the previous model resulted in scores with AUCs of 0.84 (95%-CI: 0.78–0.90), 0.83 (95%-CI: 0.77–0.89), and 0.84 (95%-CI: 0.79–0.89), respectively. Adding pallor resulted in the highest NRI for both cut-off values >1 and >2 of 4.58% (95%-CI: -3.41–12.57) and 5.18% (95%-CI: -3.91–14.28), respectively.

The final BqSOFA (consisting of the variables RR (90th percentile), CRT, BCS, and pallor) had an NRI of 51.87% (95%-CI: 40.8–63.0) and 15.5% (95%-CI: 2.65–27.5) compared to the LqSOFA for cut-off values of >1 and >2, respectively (Table G in S1 Text).

In the development cohort, 190/453 (41.9%) included children had a BqSOFA of >1. Mortality increased for each level of BqSOFA (0–4), which was not the case for LqSOFA (Fig 1). The sensitivity for a BqSOFA>1 was 91.2% (95%-CI: 81.1–96.4) and specificity 66.8% (95%-CI: 61.8–71.4, Table 2).

### Comparison BqSOFA and LqSOFA validation cohort

**Fig 3:**
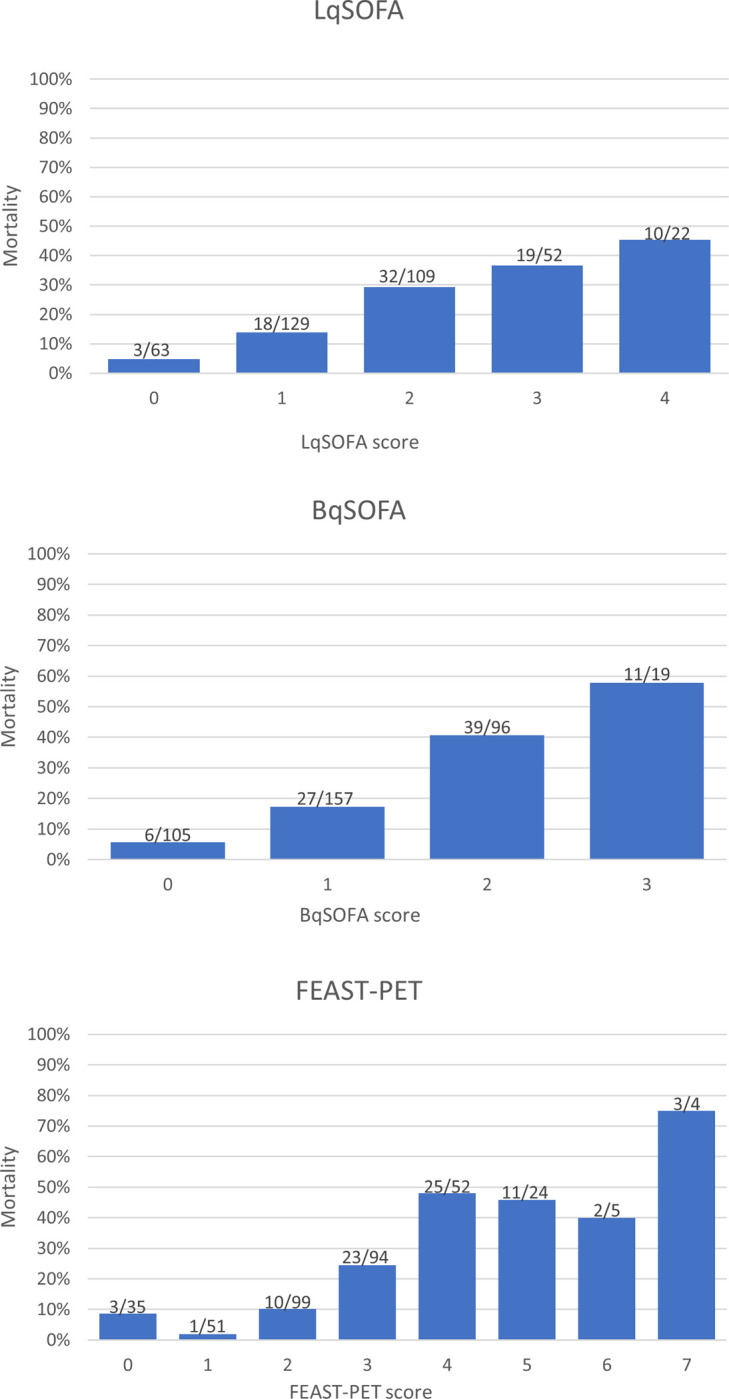
Bar charts of LqSOFA, BqSOFA and FEAST-PET in the validation cohort.

**Fig 4:**
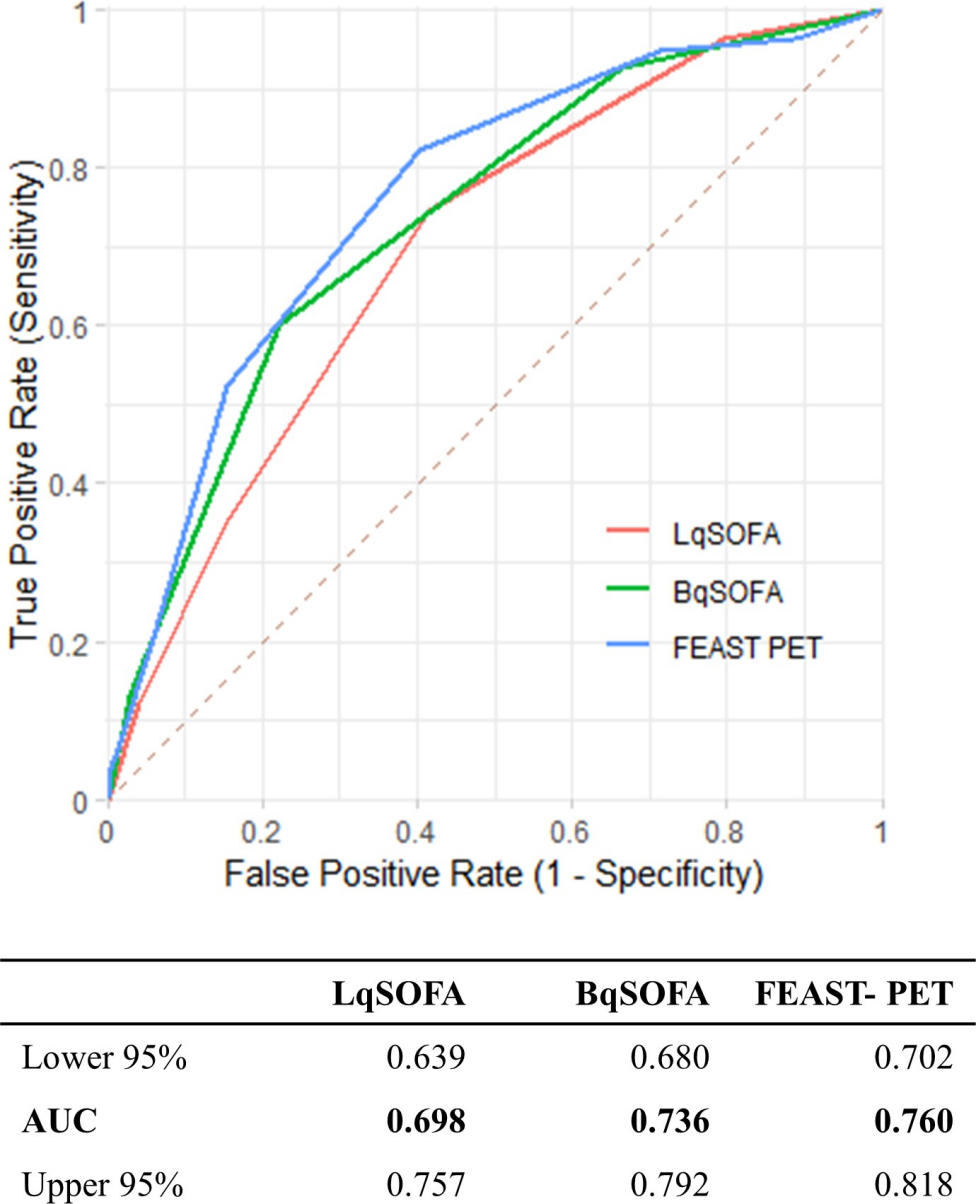
Area under receiver operating characteristic curve (AUC) for each score for predicting mortality in the validation cohort.

In the validation cohort, 312/375 (83.2%) included children had a LqSOFA>1 and 272/377 (72.1%) had a BqSOFA>1. Both scores showed higher mortality with increasing values (0–4). (Fig 3). Discriminative ability (AUC) was 0.70 (95%-CI: 0.64–0.76) for the LqSOFA and 0.74 (95%-CI: 0.68–0.79) for the BqSOFA (Fig 4). For BqSOFA>1 we found a sensitivity of 92.8% (95%-CI: 84.9–97.3) and specificity of 33.7% (95%-CI: 28.3–39.4, Table 3). The BqSOFA had an NRI of 9.65% (95%-CI: 3.95–15.36) and 4.82% (95%-CI: -4.74–14.38) compared to the LqSOFA for cut-off values of >1 and >2, respectively.

**Table 3:** Prognostic performance of LqSOFA, BqSOFA and FEAST-PET in the validation cohort.

Score	Sensitivity % (95%-CI)	Specificity % (95%-CI)	PPV^1^% (95%-CI)	NNP^2^% (95%-CI)
LqSOFA ≥1	96.3 (89.7–99.2)	20.5 (16.0–25.6)	25.3 (20.6–30.5)	95.2 (86.7–99.0)
LqSOFA ≥2	74.4 (63.6–83.4)	58.4 (52.5–64.1)	33.3 (26.6–40.7)	89.1 (83.8–93.1)
LqSOFA ≥3	35.4 (25.1–46.7)	84.6 (80.0–88.6)	39.2 (28.0–51.2)	82.4 (77.6–86.5)
BqSOFA >1	92.8 (84.9–97.3)	33.7 (28.3–39.4)	28.3 (23.0–34.1)	94.3 (88.0–97.9)
BqSOFA >2	60.2 (48.9–70.8)	77.9 (72.7–82.5)	43.5 (34.3–53.0)	87.4 (82.8–91.2)
BqSOFA >3	13.3 (6.8–22.5)	97.3 (94.7–98.8)	57.9 (33.5–79.7)	79.9 (75.4–83.9)
FEAST-PET >1	96.2 (89.2–99.2)	11.2 (7.8–15.4)	22.8 (18.4–27.7)	91.4 (0.77–98.2)
FEAST-PET >2	94.9 (87.4–98.6)	28.7 (23.5–34.3)	26.6 (21.5–32.2)	95.3 (88.5–98.7)
FEAST-PET >3	82.1 (71.7–89.8)	59.8 (53.9–65.5)	35.8 (28.7–43.2)	92.4 (87.6–95.8)
FEAST-PET >4	52.6 (40.9–64.0)	84.6 (79.9–88.6)	48.2 (37.3–59.3)	86.7 (82.2–90.5)

^1^ Positive predictive value.

^2^ Negative predictive value.

### Comparison BqSOFA and FEAST-PET in both cohorts

We compared the performance of the FEAST-PET with the BqSOFA in both cohorts. In the development cohort, AUC of the FEAST-PET was 0.83 (95%-CI: 0.77–0.89) compared to 0.84 (95%-CI 0.79–0.89) for the BqSOFA (Fig 2). In the validation cohort, the FEAST-PET and BqSOFA had AUCs of 0.76 (95%-CI: 0.70–0.82) and 0.74 (95%-CI: 0.68–0.79), respectively (Fig 4). Tables 2 and 3 include the prognostic performance of the FEAST-PET for different cut-off values in both cohorts. BqSOFA>1 showed favourable reclassification compared to FEAST-PET>2 (Table H in S1 Text).

## Discussion

We evaluated a bedside composite score using four simple clinical parameters to identify children at highest risk of dying upon admission to a hospital with limited resources. The LqSOFA performed well in a European setting, but less so in a Malawian setting. We adapted it to create the BqSOFA, validated it in a second cohort, and demonstrated improved prognostic performance for mortality which was comparable to a more complex bedside score using eight parameters (FEAST-PET).

### LqSOFA

In a large UK cohort, the LqSOFA demonstrated good prognostic performance for critical care admission and sepsis-related mortality (AUC 0.81 and 0.87, respectively) [14]. In both Malawian cohorts, nearly all children had an elevated LqSOFA score and it showed only moderate prognostic ability for mortality (AUC 0.68 and 0.70). This reflects the need for a score that is specifically developed for critically ill children in low-resource settings. The prognostic performance of the LqSOFA in these two very different settings can be influenced by varying factors such as higher mortality, more severe disease, variation of underlying risk factors, case perception, measurement techniques and availability of critical care facilities. Mortality in the UK cohort from which it was derived was 0.08% compared to 15% and 22% in the Malawian cohorts [14]. The clinical setting, pathway, population and diagnoses in Malawi are very different from the UK, as is the applicability of a composite score. In the UK, the emphasis lies on avoiding overdiagnosis and overtreatment, while in Malawi appropriate prioritization and distribution of scarce resources is most essential. The LqSOFA seems less suitable as a discriminative tool in low-resource settings, therefore we adapted it.

### BqSOFA

The BqSOFA, like other scores, consists of four parameters assessing the main vital systems: RR (respiratory), CRT and pallor (circulatory), and BCS (neurological). In developing the BqSOFA, we amended the LqSOFA by adjusting the cut-off values for RR, omitting HR, and adding pallor. BCS was used instead of AVPU, as it is the paediatric neurological score used in sub-Saharan Africa [25,26].

We used new cut-off values for respiratory rate in three age categories that are easy to remember and apply bedside and thereby improved both the simplicity and prognostic performance of the score. The LqSOFA includes cut-off values reported by Bonafide et al. and has 13 age-categories which were derived from children hospitalized in a high-resource setting with different disease severity, aetiology, and (patho)physiology [14,19]. The WHO guideline provides cut-off values for up to five years of age only [4] and other guidelines were similarly from high-resource settings, had many categories, or even lower thresholds than Bonafide et al. [27,28].

The BqSOFA had improved overall discriminative ability for mortality in both cohorts of critically ill children in Malawi compared to the LqSOFA. A low cut-off value (BqSOFA>1) had a high sensitivity and specificity. This is important in hospitals in Malawi as children at risk of dying should not be missed, but at the same time too many “false positives” will cause waste of already scarce resources. Mortality risk increased with higher score values, and a high cut-off value (BqSOFA>3) had very high specificity.

Omitting the heart rate from the composite score improved prognostic performance for mortality, irrespective of the cut-offs used, and resulted in a simpler score. It may be surprising that HR was not associated with outcome, however other studies have shown similar yet conflicting results and as a consequence HR is inconsistently included in composite scores [11,21]. This may be an example of how patient populations in low-resource settings pose specific challenges that still are not completely understood. One explanation could be the higher prevalence of severe disease and/or circulatory failure in our and other cohorts, or underlying aetiologies such as severe dehydration or severe anaemia, in which HR might not have a good discriminative ability for mortality. Another explanation might be raised intracranial pressure in critically ill children with cerebral malaria or meningitis, and HR is not increased as would be expected.

In the original qSOFA for adults, hypotension is used as the circulatory parameter, which was replaced by HR and CRT in the LqSOFA based on evidence in children [14,29]. In our setting, CRT was indeed associated with poor outcome. We added pallor to the BqSOFA as it was associated with mortality and further improved the prognostic accuracy. This association is in line with several other studies and predictive models, including the ETAT guidelines, which include pallor [4,6,16,30]. In addition to being a circulatory parameter, pallor may also reflect (severe) anaemia in children which is highly prevalent in sub-Saharan Africa and associated with increased mortality [31].

### Limitations

Limitations of this study include that the cohorts were single centre, relatively small and may represent a more critically ill population than all children admitted to emergency departments. Although there are several similarities between the two cohorts, some differences that could affect the performance should be noted. Firstly, the validation cohort was recruited 20 years ago. Since then, HIV prevalence, severe malnutrition and overall childhood mortality have reduced for various reasons including changes in immunization schedules and improved hospital protocols and resources. Secondly, different inclusion criteria applied to the cohorts, as one focused on shock and the other studied invasive bacterial infections. Although in-hospital mortality was similar over time and between the cohorts, this may have reduced the performance in the validation cohort, as more specific parameters for invasive bacterial infections (meningitis or pneumonia) were not included in the model. Lastly, the validation cohort used vital signs on admission to the ward, after initial resuscitation, which may have improved some parameters and reduced the discriminative ability of the BqSOFA. Although more similar validation populations could have been chosen, the good performance of the BqSOFA score in both cohorts underlines the robustness of the model and shows it may also be applicable as a monitoring tool in the wards after initial resuscitation.

Furthermore, 41/483 (8.3%) of children in the development cohort were excluded due to two or more missing variables needed to calculate the LqSOFA. Mortality in these children was higher (31.7%) compared to the included development cohort (13.9%). This might be an indication of severity of disease of excluded children in whom emergency care was prioritized over documentation of vital signs, which is also found in previous studies [8,32]. Other limitations include retrospective analysis of data and missing data for some clinical parameters such as RR. Finally, our main outcome was in-hospital mortality, in which we did not differentiate between death early (<48 hours) or late during admission.

### Potential practical applications and future perspectives

The BqSOFA is a simple score that can distinguish between children at high or lower risk of dying after initial triage in the emergency department. It can be calculated quickly without medical equipment, laboratory results or extensive training, unlike other previously developed scored such as SICK [7], ITAT [8], PEWS-RL [9], LODS [10], and PEDIA [6]. The FEAST-PET was developed with the same goal, to identify children with highest risk of dying upon admission, and yielded similarly good discriminative ability in our cohorts. The advantage of the BqSOFA compared to the FEAST-PET is that it consists of fewer and simpler to score parameters. The BqSOFA has a scale from 0–4 which indicates incremental risk of in-hospital mortality and may be used to prioritize critical care, if available, within the emergency department and upon admission. Although the cohorts differ in underlying aetiology and mortality of included children, the BqSOFA performs well in both cohorts and therefore shows potential for other applications in low-resource settings. We have carefully considered the design to fit the needs for low-resource settings: simplicity, limited number of false positives and aimed at interventions that can be done in a low-resource setting [33]. However, implementation strategies are key to successfully impact outcome in our and other (low-resource) settings. Future studies should focus on validation in a broader population likely to have sepsis, in emergency departments in other low-resource settings, and explore possible implementation strategies including action algorithms and impact assessment.

## Conclusion

We adapted the LqSOFA score for use in paediatric populations in a low-resource setting and validated the use of this rapid and simple bedside composite score to identify critically ill children at risk of dying upon admission to hospital in Malawi. The BqSOFA revealed improved prognostic performance for mortality compared to the LqSOFA and its performance is as discriminative yet more practical than existing scores such as the FEAST-PET. If these results can be confirmed in other low-resource settings, the BqSOFA may be used to guide level of care and focus limited resources to those needing them most.

## Supporting information

S1 Checklist Inclusivity in global research. (DOCX)

S1 Text Table A: Inclusion criteria of the development and validation cohorts Table B: Components of the LqSOFA Table C: Cut-off values for heart and respiratory rate in different guidelines Table D: Components of the FEAST-PET Table E: Components of the BqSOFA Table F: Univariate analysis of (possible) predictors for in-hospital mortality in the development cohort Table G: Stepwise approach amending the LqSOFA to develop the BqSOFA Table H: BqSOFA compared to FEAST-PET (Net Reclassification Index) Fig A: Flow diagram included children in development and validation cohort Fig B: Comparison of cut-off values for heart rate and respiratory rate in different guidelines and studies
(DOCX)
