# Supplementary material for: Identifying critically ill children in Malawi: A modified qSOFA score for low-resource settings
Source: PLOS Glob Public Health. 2024 Jan 25;4(1):e0002388. doi: 10.1371/journal.pgph.0002388 (PMC10810502; doi:10.1371/journal.pgph.0002388)
Supplement: S1 Text — Table A: Inclusion criteria of the development and validation cohorts Table B: Components of the LqSOFA Table C: Cut-off values for heart and respiratory rate in different guidelines Table D: Components of the FEAST-PET Table E: Components of the BqSOFA Table F: Univariate analysis of (possible) predictors for in-hospital mortality in the development cohort Table G: Stepwise approach amending the LqSOFA to develop the BqSOFA Table H: BqSOFA compared to FEAST-PET (Net Reclassification Index) Fig A: Flow diagram included children in development and validation cohort Fig B: Comparison of cut-off values for heart rate and respiratory rate in different guidelines and studies (DOCX) [file pgph.0002388.s002.docx]

**Table of contents**

Table A: Inclusion criteria of the development and validation cohorts 2

Table B: Components of the LqSOFA 2

Table C: Cut-off values for heart and respiratory rate in different guidelines 3-4

Table D: Components of the FEAST-PET 4

Table E: Components of the BqSOFA 4

Table F: Univariate analysis of (possible) predictors for in-hospital

mortality in the development cohort 5

Table G: Stepwise approach amending the LqSOFA to develop the BqSOFA 6

Table H: BqSOFA compared to FEAST-PET (Net Reclassification Index) 7

Fig A: Flow diagram included children in development and validation cohort 8

Fig B: Comparison of cut-off values for heart rate and respiratory rate in
different guidelines and studies 9

**Table A: Inclusion criteria of the development and validation cohorts**

| **Development cohort** | **Validation cohort** |
| --- | --- |
| Respiratory distress and/or impaired consciousness, and at least one sign of shock:   - Capillary refill time >3 seconds - Cold peripheries - Weak radial pulse - Severe tachycardia defined as:   - >180 beats per minute (bpm) if <12 months   - >160 bpm if 1-<5 years   - >140 bpm if 5-<12 years   - >120 bpm if 12-<16 years | Possible pneumonia:   - Increased respiratory rate defined as:   - >50 breaths if <12 months of age   - >40 breaths per minute if >12 months of age   OR  Meningitis:   - Stiff neck - Bulging fontanelle - Fever - Convulsions |

**Table B: Components of the LqSOFA^1^**

| Variables | Points allocated | |
| --- | --- | --- |
|  | 0 points | 1 point |
| Capillary refill time | <3 seconds | >3 seconds |
| AVPU* | A | VPU |
| Heart rate | ≤99^th^ centile Bonafide et al.^2^ age-specific thresholds | >99^th^ centile Bonafide et al.^2^ age-specific thresholds |
| Respiratory rate | ≤99^th^ centile Bonafide et al.^2^ age-specific thresholds | >99^th^ centile Bonafide et al.^2^ age-specific thresholds |

*AVPU scale: Alert, Voice, Pain, Unresponsive

1. Romaine ST, Potter J, Khanijau A, McGalliard RJ, Wright JL, Sefton G, et al. Accuracy of a Modified qSOFA Score for Predicting Critical Care Admission in Febrile Children. Pediatrics. 2020;146(4).

2. Bonafide CP, Brady PW, Keren R, Conway PH, Marsolo K, Daymont C. Development of heart and respiratory rate percentile curves for hospitalized children. Pediatrics. 2013;131(4):e1150-7.

**Table C: Cut-off values for heart and respiratory rate in different guidelines**

|  | **Age** | **WHO^1^** | **SEPSIS^2^** | **BONAFIDE 99-1^3^** | **BONAFIDE 99-2^4^** | **NICE^5^** | **P90^6^** | **FEAST PET^7^** |
| --- | --- | --- | --- | --- | --- | --- | --- | --- |
| Heart rate (beats per minute) | 0-<3  months | 160 | 180 | 186 | 180 | 160 | 205 | Upper limit: 220  Lower limit-1: 104  Lower limit-2: 79 |
|  | 3-<6 months | 160 | 180 | 182 | 180 | 160 | 205 |  |
|  | 6-<9 months | 160 | 180 | 178 | 180 | 160 | 205 |  |
|  | 9-<12 months | 160 | 180 | 176 | 180 | 160 | 205 |  |
|  | 12-<18 months | 150 | 180 | 173 | 170 | 150 | 190 |  |
|  | 18-<24 months | 150 | 180 | 170 | 170 | 150 | 190 |  |
|  | 2-<3 years | 150 | 140 | 167 | 170 | 150 | 190 |  |
|  | 3-<4 years | 140 | 140 | 164 | 160 | 140 | 190 |  |
|  | 4-<5 years | 140 | 140 | 161 | 160 | 140 | 190 |  |
|  | 5-<6 years | 140 | 140 | 161 | 160 | 130 | 175 |  |
|  | 6-<8 years |  | 130 | 155 | 150 | 120 | 175 |  |
|  | 8-<12 years |  | 130 | 147 | 150 | 115 | 175 |  |
|  | 12-<13 years |  | 130 | 138 | 140 | 130 | 175 |  |
|  | 13-<15 years |  | 110 | 138 | 140 | 130 | 175 |  |
|  | 15-<18 years |  | 110 | 132 | 140 | 130 | 175 |  |
|  | **Age** | **WHO** | **SEPSIS** | **BONAFIDE 99-1** | **BONAFIDE 99-2** | **NICE** | **P90** |  |
| Respiratory rate (breaths per minute) | 0-<1 week | 60 | 50 | 76 | 75 | 60 | 80 |  |
|  | 1 week-<1 month | 60 | 40 | 76 | 75 | 60 | 80 |  |
|  | 1-<2 months | 60 | 34 | 76 | 75 | 60 | 80 |  |
|  | 2-<3 months | 50 | 34 | 76 | 75 | 60 | 80 |  |
|  | 3-<6 months | 50 | 34 | 71 | 75 | 60 | 80 |  |
|  | 6-<9 months | 50 | 34 | 67 | 65 | 60 | 80 |  |
|  | 9-<12 months | 50 | 34 | 63 | 65 | 60 | 80 |  |
|  | 12-<18 months | 40 | 34 | 60 | 55 | 50 | 75 |  |
|  | 18-<24 months | 40 | 34 | 57 | 55 | 50 | 75 |  |
|  | 2-<3 years | 40 | 22 | 54 | 55 | 50 | 75 |  |
|  | 3-<4 years | 40 | 22 | 52 | 50 | 40 | 75 |  |
|  | 4-<5 years | 40 | 22 | 50 | 50 | 40 | 75 |  |
|  | 5-<6 years |  | 22 | 50 | 50 | 29 | 65 |  |
|  | 6-<8 years |  | 18 | 46 | 45 | 27 | 65 |  |
|  | 8-<12 years |  | 18 | 41 | 45 | 25 | 65 |  |
|  | 12-<13 years |  | 18 | 35 | 35 | 25 | 65 |  |
|  | 13-<15 years |  | 14 | 35 | 35 | 25 | 65 |  |
|  | 15-<18 years |  | 14 | 32 | 35 | 25 | 65 |  |

^1^ WHO, World Health Organisation: Pocket book of hospital care for children: guidelines for the management of common illnesses with limited resources: World Health Organization; 2005

^2^ Sepsis, Surviving Sepsis Campaign guidelines: Goldstein B, Giroir B, Randolph A. International pediatric sepsis consensus conference: definitions for sepsis and organ dysfunction in pediatrics. Pediatr Crit Care Med. 2005;6(1):2-8

^3^ Bonafide 99-1: 99^th^ percentile cut-off values: Bonafide CP, Brady PW, Keren R, Conway PH, Marsolo K, Daymont C. Development of heart and respiratory rate percentile curves for hospitalized children. Pediatrics. 2013;131(4):e1150-7

^4^ Bonafide 99-2: Simplified 99^th^ percentile cut-off values: Bonafide CP, Brady PW, Keren R, Conway PH, Marsolo K, Daymont C. Development of heart and respiratory rate percentile curves for hospitalized children. Pediatrics. 2013;131(4):e1150-7

^5^ NICE, National Institute for Health and Care Excellence: Sepsis: recognition, diagnosis and early management: © NICE (2017) Sepsis: recognition, diagnosis and early management. BJU Int. 2018;121(4):497-514

^6^ P90, 90^th^ percentile of the heart rate and respiratory rate in the development cohort: Kumwenda M, Assies R, Chatima UG, Koffi H, van Woensel JBM, Chimalizeni Y, et al. Prevalence, mortality and aetiology of paediatric shock in Malawi: a cohort study. Submitted in parallel to this paper.

^7^ FEAST PET, FEAST Paediatric Emergency Triage (PET) score: George EC, Walker AS, Kiguli S, Olupot-Olupot P, Opoka RO, Engoru C, et al. Predicting mortality in sick African children: the FEAST Paediatric Emergency Triage (PET) Score. BMC Med. 2015;13:174

**Table D: Components of the FEAST-PET**

| **Variables** | **Score given if present** |
| --- | --- |
| Axillary temperature ≤37°C | 1 |
| Heart rate <80 bpm* | 2 |
| Heart rate ≥80 to <105 bpm | 1 |
| Heart rate ≥220 bpm | 2 |
| Capillary refill time >3 seconds | 1 |
| Consciousness level: prostrate** | 1 |
| Consciousness level: coma*** | 2 |
| Respiratory distress | 1 |
| Lung crepitations | 1 |
| Severe pallor | 1 |
| Weak pulse | 1 |

* bpm= beats per minute
** Defined as Blantyre Coma Scale 3-4 and/or lethargy
*** Defined as Blantyre Coma Scale ≤2

**Supplementary Table E: Components of the BqSOFA**

| Variables | Points allocated | |
| --- | --- | --- |
|  | 0 points | 1 point |
| Capillary refill time | ≤3 seconds | >3 seconds |
| Consciousness level* | BCS =5 | BCS <5 |
| Respiratory rate | ≤90^th^ centile age-specific thresholds | >90^th^ centile al. age-specific thresholds** |
| Pallor | No | Yes |

*BCS= Blantyre Coma Scale
** Respiratory rate >80/minute if 0-1 years, >75/minute if >1-5 years, >65 if >5 years

**Supplementary Table F: Univariate analysis of (possible) predictors for in-hospital mortality in the development cohort**

| Predictor | Definition | Mortality n/N (%) | P value* |
| --- | --- | --- | --- |
| Respiratory rate | ≤99^th^ centile Bonafide et al. | 26/166 (15.7) | 0.705 |
|  | >99^th^ centile Bonafide et al | 17/121 (14.0) |  |
| Respiratory rate | ≤90^th^ centile age-specific thresholds | 33/254 (13.0) | 0.017* |
|  | >90^th^ centile age-specific thresholds | 10/33 (30.3) |  |
| Heart rate | ≤99^th^ centile Bonafide et al. | 40/183 (21.9) | <0.001 |
|  | >99^th^ centile Bonafide et al. | 21/265 (7.9) |  |
| Heart rate | ≤90^th^ centile age-specific thresholds | 56/394 (14.2) | 0.320 |
|  | >90^th^ centile age-specific thresholds | 5/54 (9.3) |  |
| Capillary refill time | <3 seconds | 29/366 (7.9) | <0.001 |
|  | >3 seconds | 33/64 (51.6) |  |
| Blantyre Coma Scale | 5 points | 24/338 (7.1) | <0.001 |
|  | <5 points | 38/108 (35.2) |  |
| Pallor | No | 35/351 (10.0) | <0.001 |
|  | Yes | 27/88 (30.7) |  |
| Nutritional status | Good or fair | 44/389 (11.3) | <0.001* |
|  | Poor | 13/34 (38.2) |  |
| Dehydration | No | 35/371 (9.4) | <0.001 |
|  | Yes | 27/62 (43.5) |  |

* χ^2^-test, Fisher exact test if sample <5 in table indicated with *

**Supplementary Table G: Stepwise approach amending the LqSOFA to develop the BqSOFA**

| **Model** | **AUC (95% CI)** | **NRI cut-off >1 for both scores % (95% CI)** | **NRI cut-off >2 for both scores % (95% CI)** |
| --- | --- | --- | --- |
| **A** | 0.793  (0.727-0.858) | NRI comparing model A to LqSOFA  40.18 (30.98-49.38) | NRI comparing model A to LqSOFA: 9.41 (-0.55-19.37) |
| **B** | 0.817  (0.756-0.877) | NRI comparing model B to A  8.18 (3.79-12.58) | NRI comparing model B to A  -0.86 (-5.51-3.79) |
| **C1 dehydration** | 0.839  (0.782-0.895) | NRI comparing model C1 to B  0.63 (-0.61-1.87) | NRI comparing model C1 to B  10.63 (1.57-19.69) |
| **C2 nutritional status** | 0.828  (0.769-0.887) | NRI comparing model C2 to B  0.76 (-4.66-6.19) | NRI comparing model C2 to B  6.14 (-0.51-12.78) |
| **C3 pallor  = BqSOFA** | 0.835  (0.785-0.886) | NRI comparing BqSOFA to model B  4.58 (-3.41-12.57) | NRI comparing BqSOFA to model B  5.18 (-3.91-14.28) |
|  |  | NRI comparing BqSOFA to LqSOFA  51.87 (40.79-62.96) | NRI comparing BqSOFA to LqSOFA:  15.05 (2.65-27.46) |

Model A: HR (p90), RR (p90), CRT, BCS
Model B: RR (p90), CRT, BCS
Model C1: RR (p90), CRT, BCS + dehydration
Model C2: RR (p90), CRT, BCS + nutritional status
Model C3: RR (p90), CRT, BCS + pallor (=BqSOFA)

**Supplementary Table H: BqSOFA compared to FEAST-PET (Net Reclassification Index)**

| **Development cohort** NRI (95% CI) | **Validation cohort**  NRI (95% CI) |
| --- | --- |
| - BqSOFA >1 vs FEAST-PET >2 18.98 (9.51-28.44) | - BqSOFA >1 vs FEAST-PET >2 3.03 (-4.73-10.79) |
| - BqSOFA >2 vs FEAST-PET >3  -13.15 (-25.27- -10.28) | - BqSOFA >2 vs FEAST-PET >3  -3.61 (-14.84-7.61) |

***
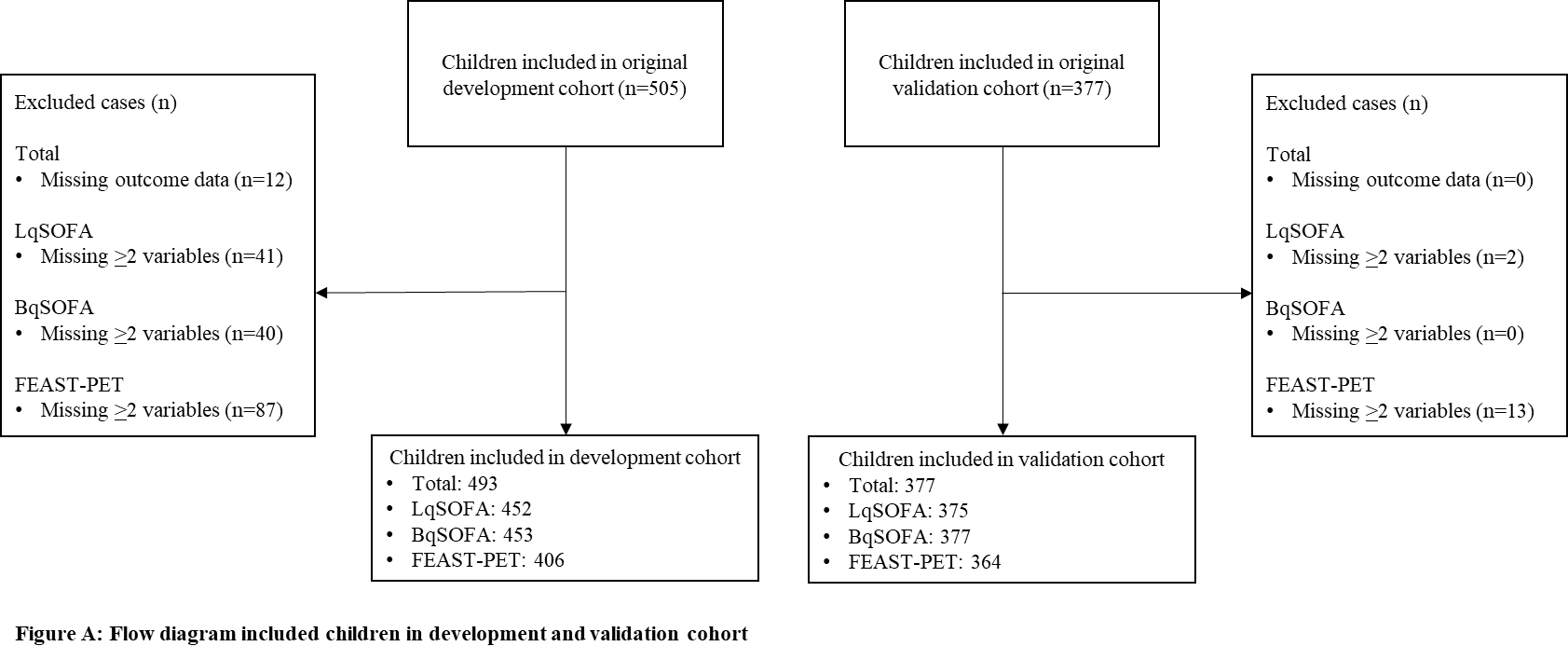
***


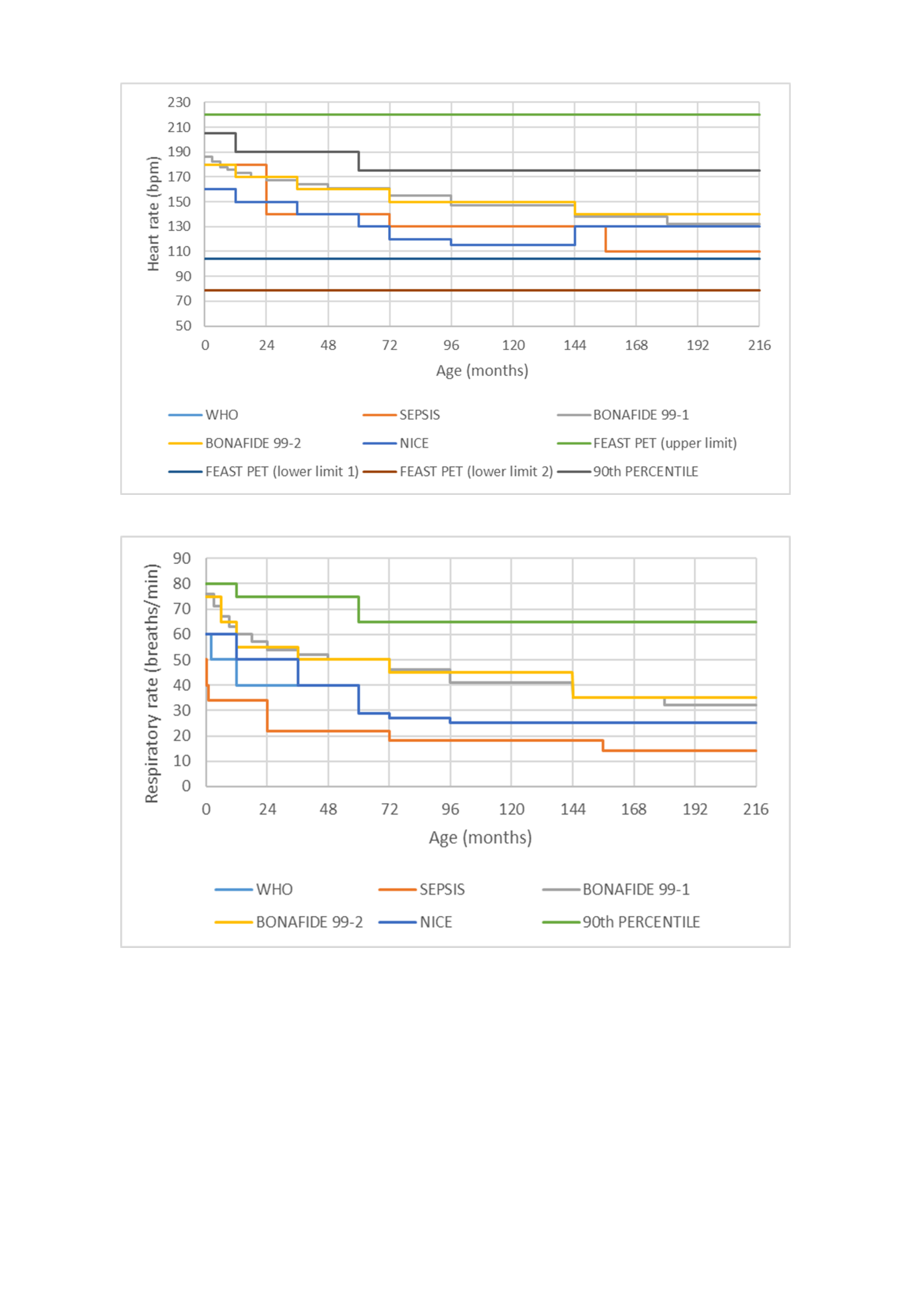
**Figure B: Comparison of cut-off values for heart rate and respiratory rate in different guidelines and studies**
